# The New-Fangled Mind Cure Craze

**Published:** 1886-06

**Authors:** 


### THE NEW-FANGLED MIND CURE CRAZE.

The eminent physiologist, lecturer and author, Prof. Joseph Rhodes Buchanan, in a recent public lecture, which consisted mainly of an exposition of the absurdities of what is usually termen "Christian healing" as amplified in a published work by Mary Barker Glover Eddy, entitled "Science and Health," holds the following language.

"The practical part of this theory, which is all that has any common sense, was developed thirty years ago by Doctor Quimby of Portland, when in practicing mesmerism and clairvoyance he found that like Professor Carpenter and others, he controlled his patient's mind, making him feel himself well, and thereby restoring his health, which he called his short-hand method.

"Long ago Mrs. Perfectsaint became his patient and was cured by him. Of course she got hold of the theory and practice, which was then honestly practiced, and when Dr. Quimby died and his companion and follower, Dr. Dresser, decided that he would not take up the mantle of the departed. Mrs. Perfectsaint discovered that by dressing up this theory as Christian doctrine and joining the warfare of Christian bigots against Spiritualism, she could enter a golden bonanza. Hence she speedily transformed herself from a Quimby patient into a Boston Messiah, and patching together a pantheistic theory, she had the hardihood to call it Christian.

"In calling it Christian they are gilding refined brass, for the doctrine recognizes neither bodies nor souls nor diseases. Christianity and common sense do recognize bodies, souls and diseases as realities for which we must work.

The author of "Science of Health" in her ravings against mesmeric or magnetic operators and clairvoyants, shows the intense malignity of her own nature—alternately raving against magnetic practitioners for whom she proposes the punishment of death."

"I don't speak of all mind cure practitioners as bombastic theorists for some of them are highly intelligent, entirely honest, modest, benevolent and as free from quackery as the practitioners of any class. But I have very little respect for pretenders who would try to supersede all that far wiser men have learned in twenty centuries by their little modicum of knowledge and their amusing delusion, that disease has no existence, but in the mind, and therefore, that a little false thinking would dispel it by denying it or ignoring it.

When a wagon runs over you and fractures your leg, a surgeon is needed ; it is of very little use to sit down and think there is nothing the matter with the broken leg.

When a child cannot be born on account of long hindrance, and we need either a pair of forceps or a ce-areansation, to bring it into the world, it would be insane to tell the mother there is nothing the matter, and sit down to help her by thinking it is all right, until she dies. These Boston follies differ very little from the vagaries that we sometimes hear in lunatic asylums, and they help the city of Boston to acquire the reputation of having more cranks to the square mile than any other portion of the United States."

I saw not long since a melancholy illustration of the metrophynical folly. "A lady of considerable intelligence, though not of a very well balanced mind, was dying with consumption, it had been preying on her for years. Her cough was frequent and distressing. As the drowning catch at straws, she seized the metrophynical doctrine and even tried to persuade me to adopt it as a matter of *policy and money making.* With her strong will she forced her mind to assent to it. She decided that she was well; that there was nothing the matter with her. "If it will cough," said she, "let it cough, it does not affect me, I am well" Poor creature; she kept on coughing and dying by inches until in a few weeks she was placed in her coffin."

"The healing which is not medical or mechanical, is performed by the power of the soul, or as some have called it, psychodynamia.

They who are gifted in that way succeed, whether they call it spirit cure, mind cure or magnetic."

